# Editorial: Vestibular Contributions to Health and Disease, Volume II-Dedicated to Bernard Cohen

**DOI:** 10.3389/fneur.2021.760822

**Published:** 2021-10-01

**Authors:** Richard F. Lewis, Michael Strupp

**Affiliations:** ^1^Departments of Otolaryngology and Neurology and Harvard Medical School, Boston, MA, United States; ^2^Jenks Vestibular Diagnostic and Physiology Laboratories, Massachusetts Eye and Ear, Boston, MA, United States; ^3^Department of Neurology, Ludwig Maximilians University, Munich, Germany; ^4^German Center for Vertigo and Balance Disorders, Munich, Germany

**Keywords:** vestibular, physiology, pathophysiology, labyrinth, brain, disease

## Introduction

The first volume of “Vestibular Contributions to Health and Disease,” published in 2018 in *Frontiers in Neurology*, sought to “highlight some of the basic science and clinical findings that have arisen over the 1st years of the twenty-first century” with regard to central and peripheral vestibular physiology and pathophysiology (1). That collection of papers was conceived and co-edited by Bernard Cohen, M.D. (universally known as Bernie) who was the driving force behind its development and completion. Bernie had an enormous passion for this project and was jubilant when it received the Spotlight Award from *Frontiers* as the best ebook collection of the year, which led to a large, international meeting at Bernie's institution, Mount Sinai Medical School in New York City in the fall of 2019. Tragically, Bernie became ill prior to the meeting, was unable to attend it, and passed away shortly after it concluded. We (RFL and MS) and *Frontiers* felt that an appropriate memorial to Bernie's life and work would be to produce a second volume of this ebook collection in his memory and honor.

Volume II contains a number of tributes to Bernie from his colleagues and these review his scientific career in detail, so we will keep our introductory remarks relatively brief. It is difficult to overstate Bernie's importance to the vestibular field, as he was one of the giants who helped define our current understanding of the vestibular system. While his *scientific* work spanned more than five decades and covered numerous aspects of peripheral and central vestibular physiology, he is perhaps best known for his investigations on the “velocity storage” mechanism in the brain, which he elucidated in many elegant studies with his long-time collaborator Ted Raphan (2). Although velocity storage was initially identified as a mechanism that improved the brain's estimate of low-frequency angular head velocity (3), it subsequently became clear that it is a critical aspect of central vestibular function since it underlies the brain's ability to synthesize the angular velocity inputs from the semicircular canals with the gravito-inertial inputs from the otolith organs (4). This canal-otolith integration contributes significantly to our ability to estimate head motion and orientation as we move through our terrestrial environment, and may also be a key to the spatial disorientation and motion sickness experienced by patients with central or peripheral vestibular dysfunction and by astronauts in microgravity.

While he was best known for his scientific work, Bernie was an M.D. and a trained *neurologist*, and maintained a clinical practice at Mount Sinai throughout his long tenure there. Indeed, applying scientific observations to improve understanding of clinical vestibular problems was one of the hallmarks of his career. For example, Bernie was the first to demonstrate that vestibular afferent nerves could be activated using implanted electrodes (5), work which has been fundamental to the development of the vestibular prosthesis (6, 7); he became fascinated with vestibular-autonomic interactions (8) and the possibility that the aberrant orthostatic responses that can underlie fainting could be habituated using vestibular stimulation; and he sought to understand the pathophysiology of mal de debarquement syndrome (MdDS), and with his colleagues Sergei Yakushin and the late Mingjia Dai, developed the first treatment approach for this recalcitrant disorder (9) which is currently being tested in a clinical trial.

Finally, Bernie was beloved by colleagues because of his *personal* qualities. He enjoyed an extensive network of friends and collaborators in the vestibular field around the world, many of whom contributed to the first and second editions of this ebook. While we cannot speak for the numerous colleagues who worked with him for decades, one of us (RFL) had the honor and pleasure of working with Bernie for several years as a co-editor of the first edition of this ebook collection, and found that Bernie combined two rare features—-a warm humanism evidenced by his immense generosity and loyalty; and a sharp, didactic mind that was constantly active. He was a rare individual and while his many friends and colleagues miss him greatly, we also are appreciative that we were able to share part of our professional and personal lives with him.

## Section 1 – Tributes and Historical Papers

Bernie provided a heartfelt tribute (Cohen) to his long-time collaborator, Mingjia Dai, who initially come from Australia for a post-doctoral fellowship, spent his entire subsequent career working with Bernie at Mount Sinai, and died from cancer in 2019. Dai focused his research on human subjects and much of his late career was spent studying MdDS and developing a novel approach to treat patients with this intractable disorder. Guyot et al. provide a tribute to Bernie's scientific accomplishments and in particular focus on his work in the 1960's when he and Suzuki first showed that individual canal ampullary nerves could be activated with stimulation provided by electrodes implanted in the canal's ampulla (5). This work provided the foundation for the vestibular prosthesis, which has been in development for the past two-decades in animal models and has more recently moved into clinical trials (7). Maruta provides another tribute to Bernie and his scientific contributions, tracing the broad range of his research interests and contributions which touch on nearly all aspects of vestibular function. Finally, Straka et al. provides an overview of the work of Steinhausen, which previously was only available in German. They describe the pioneering work Steinhausen performed in the early 20th century in which he deduced the relationship between cupular displacement and vestibular-mediated eye movement responses.

## Section 2 – Mal De Debarquement Syndrome

Bernie focused much of his late career on mal de debarquement syndrome (MdDS), a disorder where patients continue to feel as if they are moving after they experience passive vehicular motion (10), and MdDS was therefore well-represented in Volume I of “Vestibular Disorders in Health and Disease” and at the meeting based on that volume. The three articles on MdDS in the current volume examine different aspects of this disorder's pathophysiology and potential approaches to therapy. Mucci et al. propose that neural loops between the vestibular nuclei and the cerebellar nodulus and uvula form an oscillator that cancels the postural disturbance produced by vehicular motion (e.g., in a boat) but can become pathologically potentiated and produce the persistent feeling of motion experienced by patients with MdDS. Cha et al. use functional and structural imaging in MdDS patients and found abnormalities that support aberrant sensitization of limbic structures in the brain. Based on these observations, they propose that non-invasive brain stimulation (e.g., transcranial magnetic stimulation) may be able to attenuate the synchronized oscillations in these brain regions that may underlie the persistent illusion of motion in MdDS. Yakushin et al. extend the MdDS treatment approach developed at Mount Sinai, which utilizes head motion coupled to optokinetic stimulation, to a virtual reality stimulus provided by a portable app that patients can utilize at home. In a small study, they showed promising results using this method, as all of the treated MdDS patients noted substantial symptomatic improvement after treatment.

## Section 3 – Vestibular-Autonomic Interactions

The relationship between the vestibular and autonomic nervous systems has been a relatively neglected area of vestibular physiology, and Bernie became fascinated with this topic late in his career. In particular, he sought to understand the contributions of the vestibular system to orthostatic hypotension (8) and the potential susceptibility of these interactions to therapy. The current volume contains three articles that focus on vestibular-autonomic interactions. Raphan and Yakushin, both long-time collaborators of Bernie's, applied machine learning to train their model of vasovagal responses, and with this approach defined a hyperplane that segregates normal and pathologic blood pressure responses to changes in body orientation. Rice et al. activated vertical semicircular canals using infrared light and were able to modulate autonomic behaviors with this approach, indicating that the vertical canals (and not only the otolith organs) provide sensory information that is used to regulate cardiovascular responses. Bielanin et al. recorded from brainstem regions that have been proposed to use vestibular inputs to modulate cardiovascular responses (the medullary lateral tegmental field and the nucleus solitarius tract) in awake felines and found that more than a third of neurons in these regions responded to large static head tilts, implying that these regions constitute part of the central vestibulo-sympathetic reflex pathway.

## Section 4 – Vestibular Science

Seven papers focus on other aspects of vestibular science and include physiologic and behavioral studies.

### Vestibular Physiology

Curthoys provides a tribute to Yoshio Uchino, reviewing his contributions to the understanding otolith function and its clinical testing (11). Uchino elegantly demonstrated that activating hair cells that were located on different sides of the strioloa and had opposing polarizations elicited central responses that summed rather than canceled, and that utricular signals in the brain have inhibitory commissural projections that recapitulate the “push-pull” processing of central semicircular canal signals. These observations provide a context for the clinical otolith tests that utilizes sound or vibration to activate the saccules or utricles, respectively. Barmack and Pettorossi review the vestibulo-cerebellum adaptation that occurs in response to prolonged vestibular and optokinetic stimulation, and in particular, describe the relationship between behavioral (e.g., eye movement) and molecular biologic (e.g., corticotropin releasing factor) manifestations of cerebellar adaptation that contribute to postural control.

### Vestibular-Mediated Behaviors

Two papers examine *eye movements* in normal human subjects and specifically investigate characteristics of the central velocity-to-position neural integrator initially demonstrated by Robinson (12). Ritter et al. describes the characteristics of gaze-evoked nystagmus and rebound nystagmus in people without otologic or neurologic disease. By quantifying these eye movement characteristics in normal subjects, they provide information which improves the ability to determine when these oculomotor findings are abnormal and indicative of underlying cerebellar disease. Lädrach et al. examined Alexander's law [slow phase velocity increases when the eyes are directed toward the nystagmus quick phase, (13)] in normal subjects by measuring post-rotatory nystagmus with the head upright or tilted. Their results support the hypothesis that Alexander's law results from an asymmetry in neural integrator function rather than a gradual adaptive process. Lackner reviewed a series of studies that demonstrate the effects of haptic stabilization introduced by light touch on *balance*. He posits the presence of a long-loop cortical reflex whereby sensory cues from the hand modulate motor activity in the legs and thereby reduce postural sway, and suggests that this mechanism is important for patients with vestibular damage and for astronauts experiencing microgravity. Two papers examined vestibular effects on *perception* and *cognition*. Wedtgrube et al. studied the effects of prolonged static tilt and optokinetic stimulation on the subjective postural vertical (e.g., the ability to align the body's longitudinal axis parallel to gravity). They found that prolonged tilt biases the subjective postural vertical in a manner that is consistent with Bayesian models since the “prior” is presumable shifted by the long-term static tilt. Dobbels et al. demonstrate that bilateral vestibular damage does not appear to affect performance on a virtual Morris water maze (vMWM) that is performed with the head stationary (e.g., a condition where no dynamic vestibular responses are elicited). This result contradicts those of a prior study (14) and suggests that visuo-spatial processing may not be affected by the absence of vestibular inputs. Conversely, an alternate explanation for their findings is that the vMWM is an inadequate assay of these visuo-spatial functions in human subjects.

## Section 5 – Vestibular Disorders

Eight papers examined aspects of vestibular dysfunction, including peripheral vestibular damage, central compensation, the real-world behavioral consequences of vestibular damage.

### Peripheral Disorders–Meniere's Disease

Two papers examine how vestibular testing is affected by Meniere's Disease, including the primary form of the disease (etiology unknown) and the secondary form [also called delayed endolymphatic hydrops, where an initiating event can be identified, (15)]. Leng and Liu measured caloric and video head impulse tests (vHIT) in nineteen patients with delayed endolymphatic hydrops, and while the majority of patients had abnormal caloric responses, only a small minority had abnormal horizontal canal vHIT results. They suggest that this pattern of test findings may be a distinctive feature of delayed endolymphatic hydrops. Kaci et al. reviewed the literature on vHIT testing in patients with Meniere's Disease and proposed possible pathophysiologic explanations for these findings.

### Peripheral Disorders–Other Etiologies

Three papers considered other types of peripheral vestibulopathies, including those caused by noise exposure, aging, and covid. Stewart et al. reviewed the effects of noise on vestibular function and described that noise can induce both peripheral and central vestibular damage in animals, particularly in the otolith organs. Much less is known about noise effects in humans, however, but saccular-collic reflexes appear to be damaged by noise, perhaps because the saccule can be activated by sound in a manner that is not recapitulated by other vestibular end-organs. Wagner et al. reviewed the effects of aging on vestibular dysfunction and its effects on balance. They indicate that the contribution of age-related vestibular damage to the imbalance associated with aging remains poorly understood and that further work is required elucidate this connection more accurately. Li et al. review the relationship between covid-19 and symptoms of dizziness and vertigo in China. They report that the etiology of dizziness shifted between the pre and per covid time frames, with an increase in benign positional vertigo and psychogenic dizziness, and a decrease in “vascular” vertigo. While these results are not readily explainable, they do support the contention that medical providers should be aware of these shifts when evaluating vestibular patients during the covid epidemic.

### Central Compensation After Peripheral Damage

Two papers addressed issues related to the compensation that occurs in the brain after the vestibular periphery is damage. McGarvie et al. used vHIT to assess recovery of the vestibulo-ocular reflex (VOR) in a patient with bilateral sequential vestibular neuritis over a period of 500 days. They found that VOR recovery occurred with an exponential time course that depended on the velocity of the head motion stimulus, as responses to low-velocity head rotations had a time constant of about 100 days, but responses to high-velocity rotations improved more slowly with a time constant of about 150 days. Tarnutzer et al. examined VOR changes in patients with a unilateral vestibular schwannoma if they receive intratympanic gentamicin in the ear with the tumor prior to surgical resection of the schwannoma. As predicted, the VOR gain was reduced after intratympanic gentamicin injections, but the anterior canal was largely spared for uncertain reasons. They conclude that if gentamicin injections are provided prior to vestibular schwannoma surgery, the severity of the post-surgical vestibular deficit (e.g., vertigo, imbalance) may be reduced since a more gradual and controlled reduction in vestibular function occurred prior to surgical deafferentation of one vestibular nerve.

### Real-World Consequences of Peripheral Vestibular Damage

van Leeuwen et al. provide the perspective of vestibular patients on the difficulties encountered when driving. They asked vestibular patients to complete a questionnaire about driving and from 432 responses concluded that 44% experienced difficulties driving. Furthermore, physicians rarely discussed driving issues or appropriate precautions with the vestibular patients. They opinioned that physicians should discuss driving issues with vestibular patients since these driving difficulties can limit their ability to work and perform other normal daily tasks.

## Conclusions

This second edition of “Vestibular Contributions to Health and Disease” covers a wide range of vestibular topics, including normal vestibular physiology and abnormal vestibular disease processes. More specifically, Bernie's late career interests of normal (vestibulo-autonomic) and abnormal (MdDS) vestibular topics both receive considerable attention. In conclusion, Bernie's many friends and colleagues miss him greatly and we hope that this *Frontiers* ebook collection dedicated to his memory provides a small indication of the high regard he was held by all as a scientist, a clinician, and a person.

## Author Contributions

RL and MS contributed equally to the writing of this editorial. Both authors contributed to the article and approved the submitted version.

## Conflict of Interest

The authors declare that the research was conducted in the absence of any commercial or financial relationships that could be construed as a potential conflict of interest.

## Publisher's Note

All claims expressed in this article are solely those of the authors and do not necessarily represent those of their affiliated organizations, or those of the publisher, the editors and the reviewers. Any product that may be evaluated in this article, or claim that may be made by its manufacturer, is not guaranteed or endorsed by the publisher.
